# Optical calibration and performance of the adaptive secondary mirror at the Magellan telescope

**DOI:** 10.1038/s41598-018-29171-6

**Published:** 2018-07-17

**Authors:** Runa Briguglio, Fernando Quirós-Pacheco, Jared R. Males, Marco Xompero, Armando Riccardi, Laird M. Close, Katie M. Morzinski, Simone Esposito, Enrico Pinna, Alfio Puglisi, Lauren Schatz, Kelsey Miller

**Affiliations:** 1 INAF Osservatorio Astrofisico di Arcetri, L. E. Fermi 5, 50125 Firenze, Italy; 2 GMTO, 465 N. Halstead St., Suite 250, Pasadena, CA 91107 USA; 3 Steward Observatory, Department of Astronomy, University of Arizona, Tucson, AZ 85721 USA; 4 College of Optical Science, University of Arizona, Tucson, AZ 85721 USA

## Abstract

In this paper we describe the procedure for the optical calibration of large size deformable mirrors, acting as wavefront correctors for adaptive optics systems. Adaptive optics compensate the disturbance due to the atmospheric turbulence to restore the telescope resolution. We will showcase in particular the activities performed for the Adaptive Secondary Mirror (ASM) of the Magellan Adaptive Optics system (MagAO), which is an instrument for the 6.5 m Magellan Clay Telescope, located at Las Campanas Observatory, in Chile. The MagAO ASM calibration is part of the MagAO-2K project, a major MagAO upgrade that started in 2016 with the goal of boosting adaptive optics (AO) correction at visible wavelengths to image exoplanets. For the first time, the optical quality of MagAO mirror is reported. We describe the procedures developed to achieve high SNR interferometric measurements of the ASM modes under the presence of dome convection noise and telescope vibrations. These measurements were required to produce an improved control matrix with up to 500 modes to close the AO loop on sky with enhanced performances. An updated slaving algorithm was developed to improve the control of actuators vignetted by the central obscuration. The calibrations yielded also a new ASM flattening command, updating the one in use since the MagAO commissioning in 2013. With the new flattening command, a 22 nm RMS surface error was achieved. Finally, we present on-sky results showing the MagAO performance achieved with the new calibrations.

## Introduction

Large ground-based telescopes are often equipped with Adaptive Optics (AO) systems^1^ to compensate for the wavefront distortions introduced by the atmospheric turbulence and restore, at least partially, the telescope diffraction-limited resolution. In particular, AO is an indispensable capability in those fields in astrophysics where high resolution is required, like exoplanets and protostellar disks imaging. In an AO system, a wavefront sensor (WFS) measures at high cadence the optical turbulence and sends commands to a deformable mirror (DM) acting as the wavefront corrector. The WFS and the DM are placed in a closed-loop control configuration.

While all the early AO systems and many of the current ones feature an optical relay to re-image the telescope entrance pupil on a small post-focal DM, some current telescopes have been transformed into fully adaptive ones, by replacing the conventional (rigid) secondary mirror with an adaptive one (adaptive secondary mirror, ASM). With respect to a post-focal DM, an ASM offers a number of key advantages, namely: it delivers a corrected wavefront to any focal station of the telescope, with a minimal number of optical surfaces; thermal emissivity is substantially reduced and the optical throughput is increased; a lower impact of the high spatial frequency, manufacturing errors and actuators print-through. The technology of large ASM was developed in the 2000 s, and was first demonstrated at the MMT on Mt. Hopkins, Arizona, and then implemented at the Large Binocular Telescope (LBT)^2^, in Mt. Graham, Arizona, the Magellan Clay Telescope^3^, in Las Campanas Observatory (LCO), Chile, and more recently, at the ESO VLT-UT4^4^ in Paranal Observatory, Chile. The ASM technology has been also selected for the next generation of extremely large telescopes (ELT): the Giant Magellan Telescope (GMT) will feature a 7 segments ASM^5^, while the wavefront corrector of the European ELT (E-ELT) will be a flat, deformable quaternary mirror^6^. Given the large format of the ASM technology, the same concept has been also proposed as an adaptive primary for the space telescope in the LATT project^7^. Therefore, large deformable mirrors have become a key component of major AO systems for ground-based telescopes and will likely play a role in future space telescope projects too.

All these systems share the same working principle. The optical surface is a thin glass shell (TS) 1 to 2 mm thick, with magnets bonded on its back. The actuators are voice coil motors, each facing a corresponding magnet and exerting a contactless force on it. The actuators are mounted inside a plate (called reference body, RB) behind the TS. For the system already in use at an observatory, including MagAO ASM, the RB is a thick Zerodur glass meniscus; for future systems such as the GMT and E-ELT deformable mirrors, Silicon Carbide has been considered. The RB provides a reference for position capacitive sensors co-located with the actuators. Thanks to such internal metrology, the actuator forces are controlled in a local, position closed loop by the ASM electronics. In nominal working conditions the TS *floats* in front of the RB at a given distance: such gap is 60 *μ*m for the MagAO ASM and will be larger for future systems. A larger gap allows a greater movement range for the TS, valuable to implement field stabilization and chopping mode. When the AO loop is closed, the WFS sends commands to the ASM and the actuators modify the TS surface to compensate for the wavefront aberrations.

The Magellan Adaptive Optics system (MagAO)^3^ was designed as a modification of the LBT AO systems, and specifically tailored for visible light AO imaging as demonstrated in L. Close *et al*.^8^ Similarly to the LBT AO systems, the MagAO features a pyramid WFS and a concave ASM, with a 1.6 mm thick TS. Given the particular design of the Magellan telescope, the MagAO ASM is 85 cm in diameter (≈ 5 cm less than the LBT), and is actively controlled by 585 actuators (87 actuators less than the LBT). Furthermore, due to the 0.29 central obscuration of Magellan, the first three inner rings of actuators are vignetted. This particular feature required the development of specific *slaving* algorithms to control these un-illuminated actuators (see Section 4.4).

MagAO was integrated and tested at the Arcetri Test Tower (ATT) in Italy in 2011–2012. Laboratory tests included the interferometric calibration of the ASM, the functional verification of the system and the measurement of the close loop performances with the pyramid WFS. MagAO was then commissioned at LCO in 2012–2013 and used regularly for scientific observations since then. In 2016, the MagAO-2K^9^ project started, consisting in a major upgrade of the MagAO components to boost the AO correction at visible wavelengths. These updates include an improved ASM modal calibration (the subject of this paper), improvements in WFS spatial sampling by 10% (from 27 × 27 to 30 × 30 sub-apertures across the diameter), and an increased speed of operation (from 1 to 2 kHz) to reduce latency errors.

The procedure for the optical calibration of an adaptive mirror consists in the interferometric measurement of the mirror surface when a known actuator command is applied. The procedure is basically split into two steps. At first, the actuators are excited and the associated optical responses are recorded; such shapes are the actuator influence functions (IF). As the actuators represent the degrees of freedom of the mirror, the collection of the IF (or *optical interaction matrix*) provides a convenient description of the system in terms of the optical shapes it may produce. The second step is the *mirror flattening*, i.e. the correction of the mirror surface through the application of the (negative) actuator command that best matches the current optical shape. The optical calibration at the telescope of the MagAO ASM was done for the first time in Nov 2016. The results of the calibration will be presented in Section 2. The methods and setup used for this purpose are described in Section 4.

## Results of the Optical Measurements

### Initial mirror shaping

**Figure 1:**
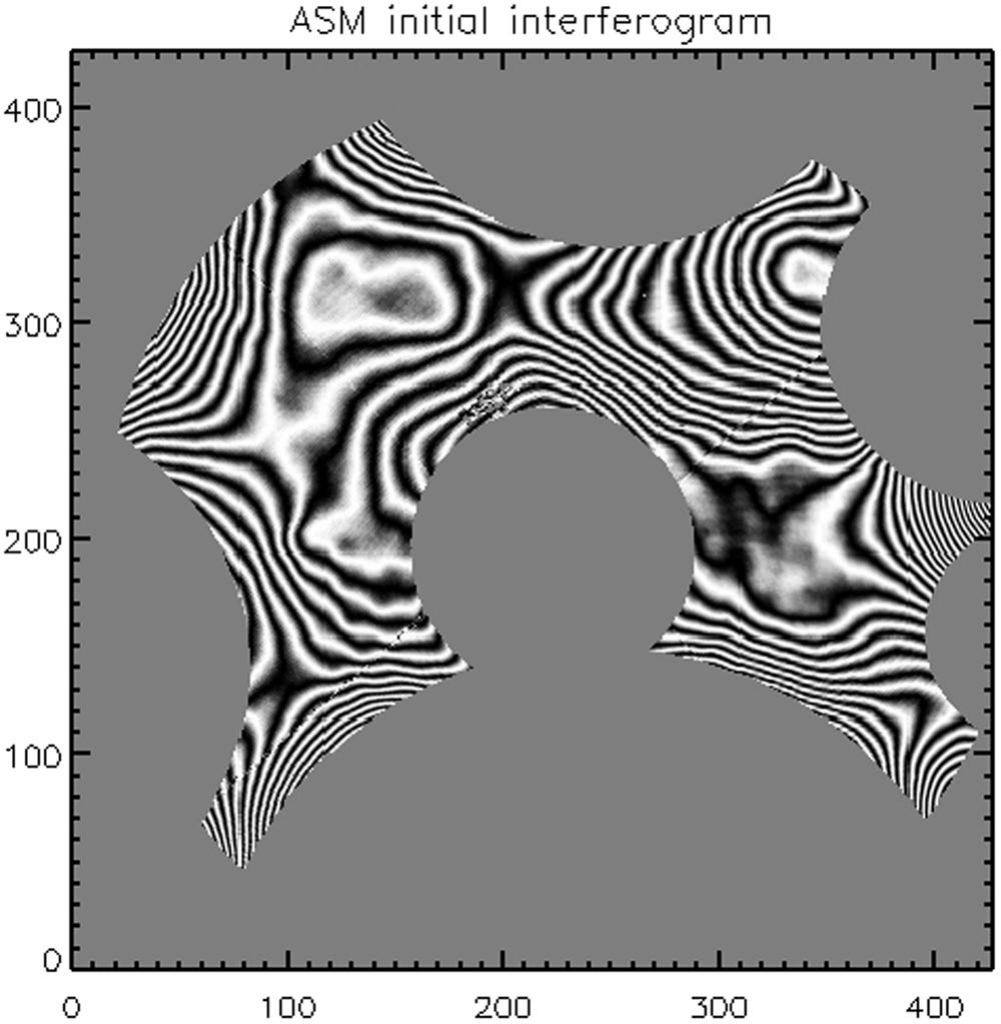
First ASM fringes image (at a wavelength of 633 nm) after alignment. The slope error on the masked areas is larger than the interferometer capture range.

**Figure 2:**
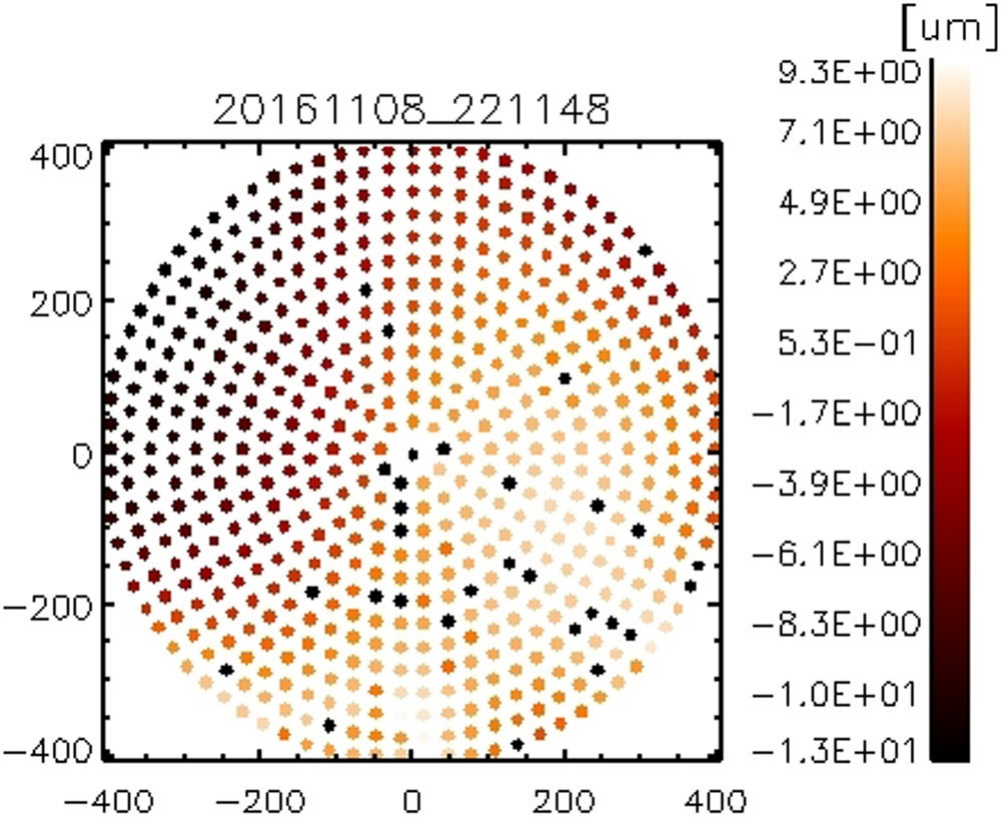
Actuator command applied for the initial ASM shaping. Isolated black dots represent not-working actuators.

**Figure 3:**
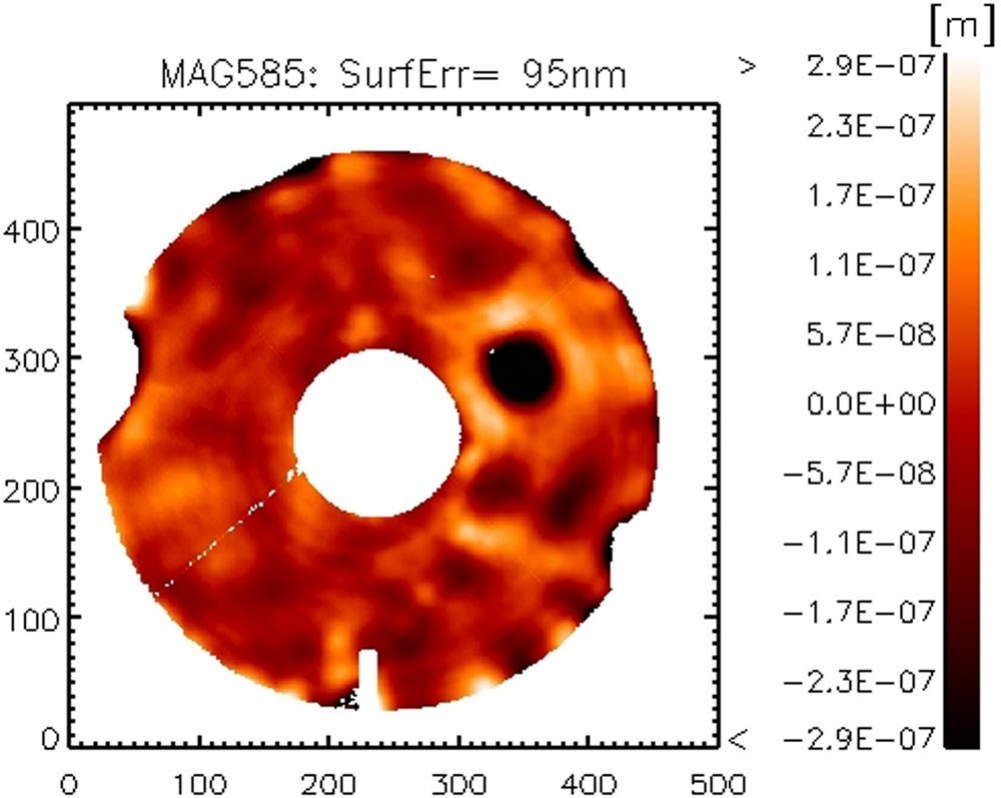
ASM surface map after the preliminary shaping. The black spot is due to a single actuator with very poor calibration at this stage. The missing areas (wrt the perfect circular pupil) are masked out because the local slope is beyond the interferometer capture range.

After the initial alignment of ASM, tertiary mirror and interferometer, we started the preliminary flattening procedure with the aim to reduce the local ASM surface slope. The initial surface map of the ASM is shown in Fig. 1, where a large fraction of the pupil lays beyond the interferometer capture range. The preliminary shaping process consists in the sequential application of low order mirror modes in a *trial and error* process with the visual feedback of the interferometer. The final command after such preliminary shaping is shown in Fig. 2; we applied a ~20 *μ*m PtV tilt and coma to correct for the offset of the preliminary alignment, plus a number of gaussian-shaped commands, centered on the most miscalibrated actuators, to improve the local visibility. The ASM figuring after such process is shown in Fig. 3, where the large surface error (SE) of 95 nm RMS is partially due to a single miscalibrated actuator (which has been corrected during the final flattening). Such initial procedure is so far executed *by hand* to optimize manually the interferometer sampling option, e.g. the detector mask; an automated mechanism may be however implemented and may be useful for segmented mirrors, where the same procedure shall be run inividually for each segment.

### Mirror flattening

The flattening procedure is based on the measurement of the IF and their correction in close loop and is fully described in Sec. 4.1. The process is iterative and a larger number of modes is measured at each step, as soon as the local surface slope is reduced. Such a strategy allows the optimization of the modal command amplitude to measure the IF with a suitable SNR without saturating the fringes density on the interferometer. We found that the use of modal IF is more efficient with respect to *local* ones, i.e. a single actuator is poked; the correction of global shapes allows a quicker convergence, as it has been experienced during the optical calibration of the LBT and VLT ASMs: in those cases, a linear combination of the first 50 to 100 mirror modes was enough to reduce the local surface slope well within the interferometer capture range and proceed with the sampling of the entire set of IF in a single shot. In addition, local IF have signal-to-noise ratio (SNR) different from 0 only in correspondence of the poked actuator.

The IF are measured with the *push - pull* differential sampling running at the maximum interferometer frame rate (25 Hz) to freeze the slowly evolving noise due to air convection; at this stage, 5 to 15 *push-pull* samples were averaged together, achieving a minimum SNR of 15 to 10 for the high spatial scales (modes 300 to 500). Such value is computed by comparing the typical modal amplitude (50 nm to 30 nm RMS) with the measurement noise shown in Fig. 8, for the selected number of *push-pull* averages.

At any step *i*, the flattening command *c*_*i*_ is computed as

1 $${c}_{i}=-\,{M}_{i}^{+}{w}_{i},$$

where ${M}_{i}^{+}$ is the pseudo-inverse of the interaction matrix at step *i* and *w*_*i*_ is the actual surface map. The flattening process consists therefore in the minimization of the SE within the current map *w*_*i*_.

**Figure 4:**
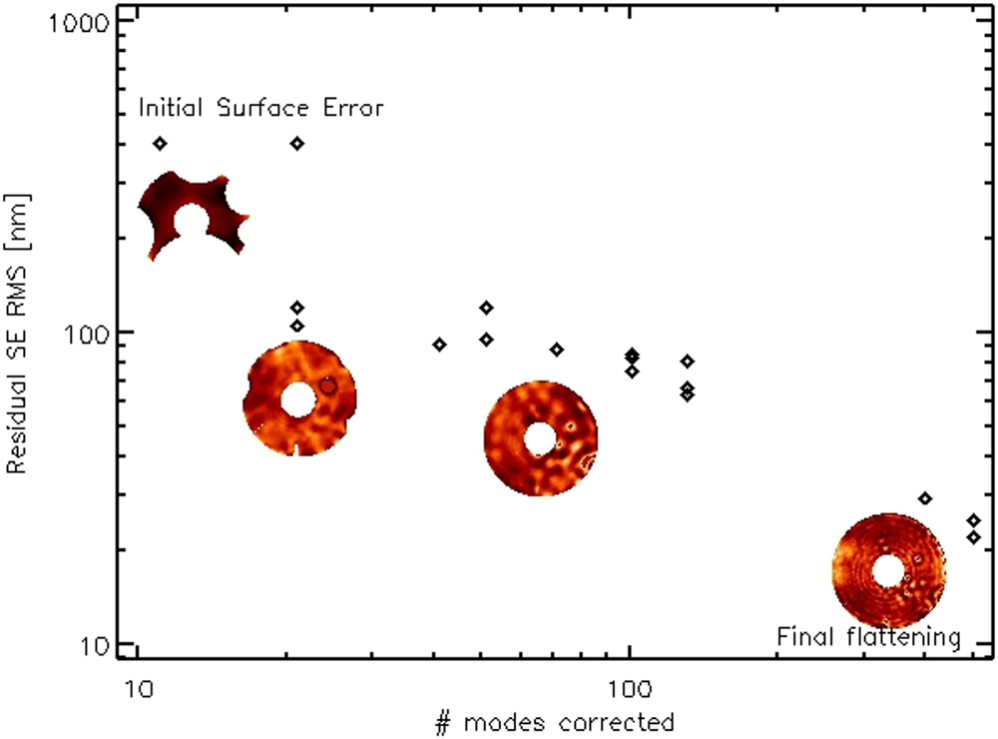
Residual surface error during the flattening process, versus number of corrected modes.

**Figure 5:**
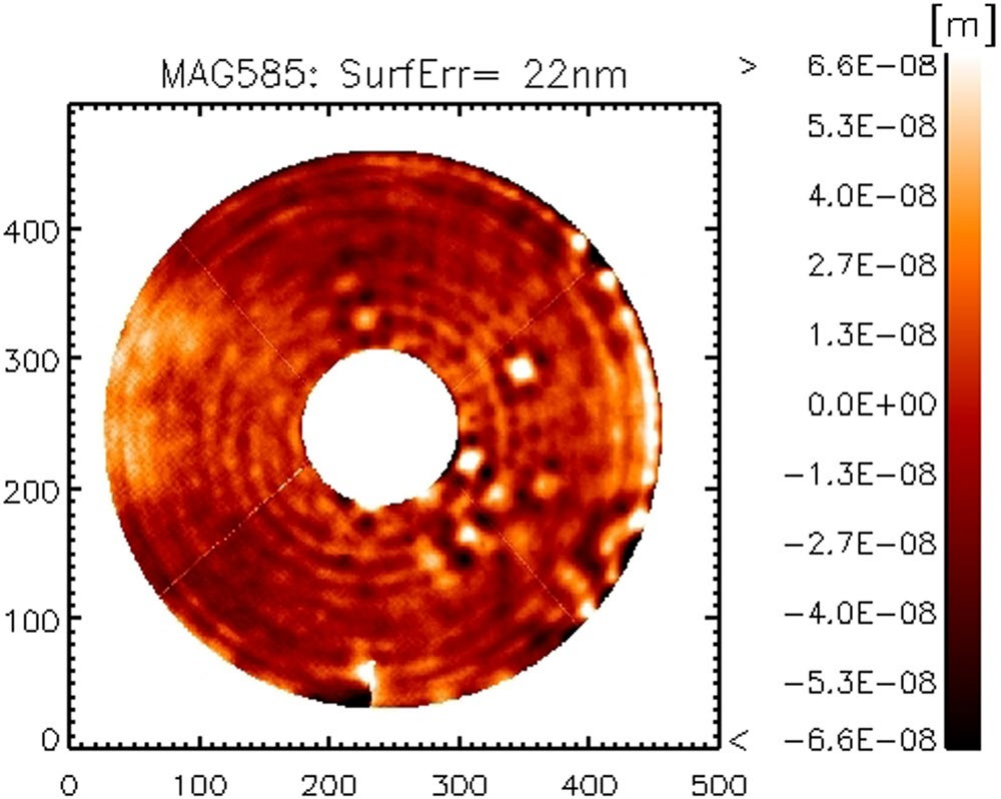
Final flattening realization after correcting 500 modes. The low order features are due to non-averaged convection noise.

The mirror modes in the range 0 to 130 have been sampled in 5 steps: at each step, we repeatedly computed and applied the flattening command to check the process convergence. After correcting 130 modes, the local slope was low enough to allow the sampling of the entire set of 555 mirror modes in a single run. The flattening result for entire run is summarized in Fig. 4, where we show the procedure is converging to a typical SE of 20 to 30 nm, after correcting 500 modes out of 555. The process was stopped at that point because of schedule reasons. The final ASM figuring is 22 nm RMS and the surface map *w*_*f*_ is shown in Fig. 5; the main contributors to the residual SE are: un-averaged air convection (the low order features), local spots due to the disabled actuators (mostly those at the edge), inter-actuator features (rings) due to stress released after correcting for focus and coma and the print-through given by magnets bonding.

The same dataset *w*_*f*_ in Fig. 5 may be further processed to compute a *synthetic flat* map, i.e. to remove from the *w*_*f*_ those features which are still within the actuator control space after the flattening process: for instance, the air convection pattern that has evolved between the flattening measurement and its verification. The procedure consists in computing the expected mirror shape

2 $${w}_{s}={w}_{f}-M{M}^{+}{w}_{f}$$

and is a valuable check for errors in the flattening process. The SE of *w*_*s*_ is 14 nm RMS and represents the theoretical flattening realization (or best flat) achievable with the number of modes in *M*. The square difference with the actual flattening value is 17 nm RMS and is mostly given by low orders content due to air convection noise. Such value is consistent with our estimation of noise, shown in Sec. 2.3.

**Figure 6:**
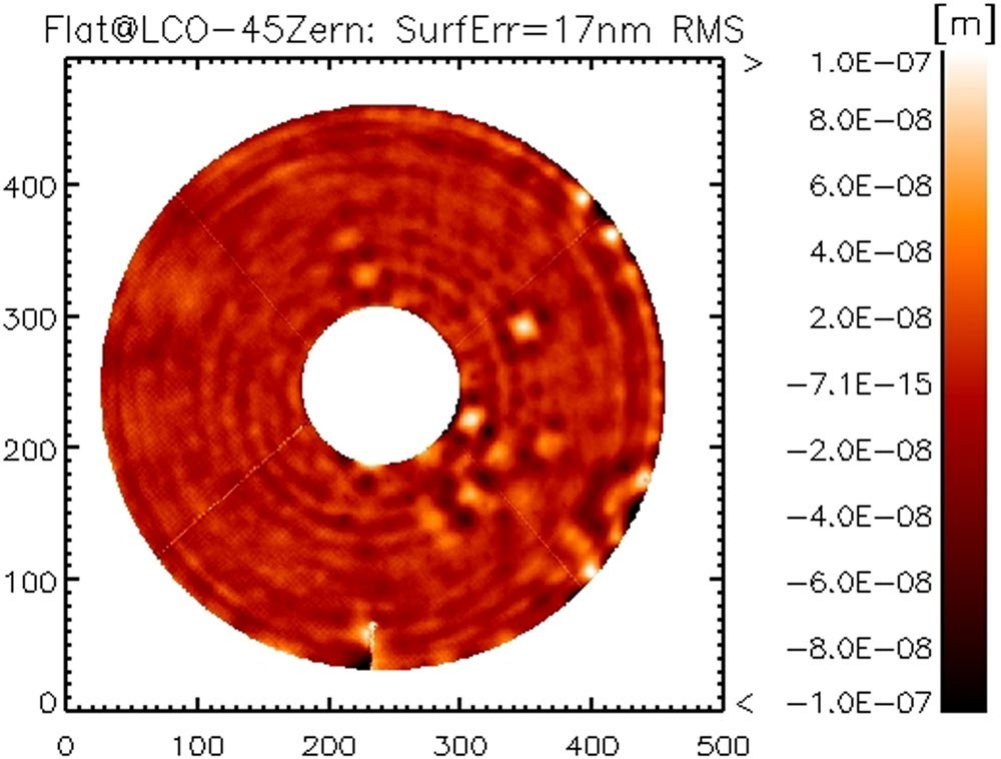
Flattening realization at LCO, 45 Zernike modes subtracted to reduce the convection noise.

**Figure 7:**
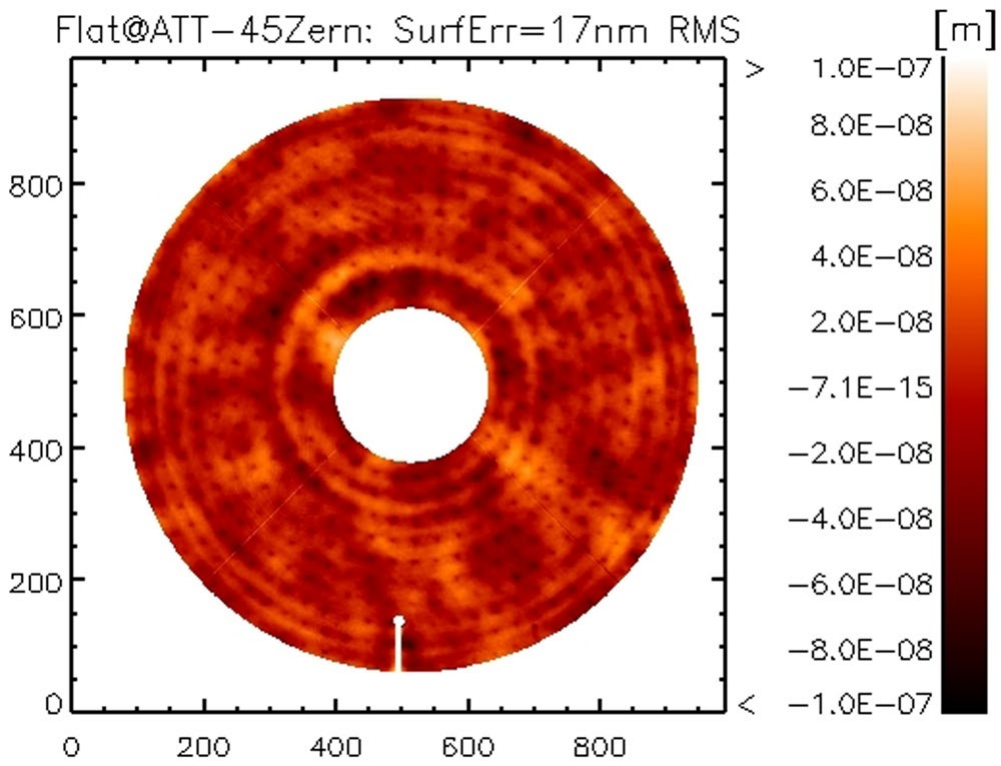
Flattening realization at ATT (2011), 45 Zernike modes subtracted to reduce the convection noise.

We compared the current result *w*_*f*_ with the one obtained on the ATT bench in Italy after the system integration and calibration in 2011. In that case we corrected 350 modes only with no robust time averaging to suppress convection noise. Both realizations are shown in Figs 6 and 7: in both cases, 45 Zernike modes have been subtracted to reduce the effect of low order features from non-averaged convection noise and allow an efficient comparison. The resulting surface error is 17 nm RMS for both cases: such result indicates also that the high orders content in the mirror shape (modes 350 to 550) is negligible.

The Strehl ratio in V band (500 nm) expected with such surface error is 99.3%.

### Dome convection noise

After flattening the mirror, we measured the noise level due to air convection inside the dome. Convection noise affects the interferometric measurements both as a precision error (for the case of the IF differential sampling) and as an accuracy error, for absolute samplings such as during the flattening process. The noise measurement procedures are described in Sec. 4.3. Estimating the noise is of crucial importance to adjust the sampling parameters, for instance to allow the precise measurement of the very low amplitude mirror modes (see also the next section) and to assess the time integration needed to achieve a given measurement offset error. We collected noise measurements at different times in the day and compared the results. We identified that the lowest convection noise is measured at noon, when the sun close to zenith heats the dome from the top, thus creating a positive vertical thermal gradient inside the chamber, and preventing the air convection.

**Figure 8:**
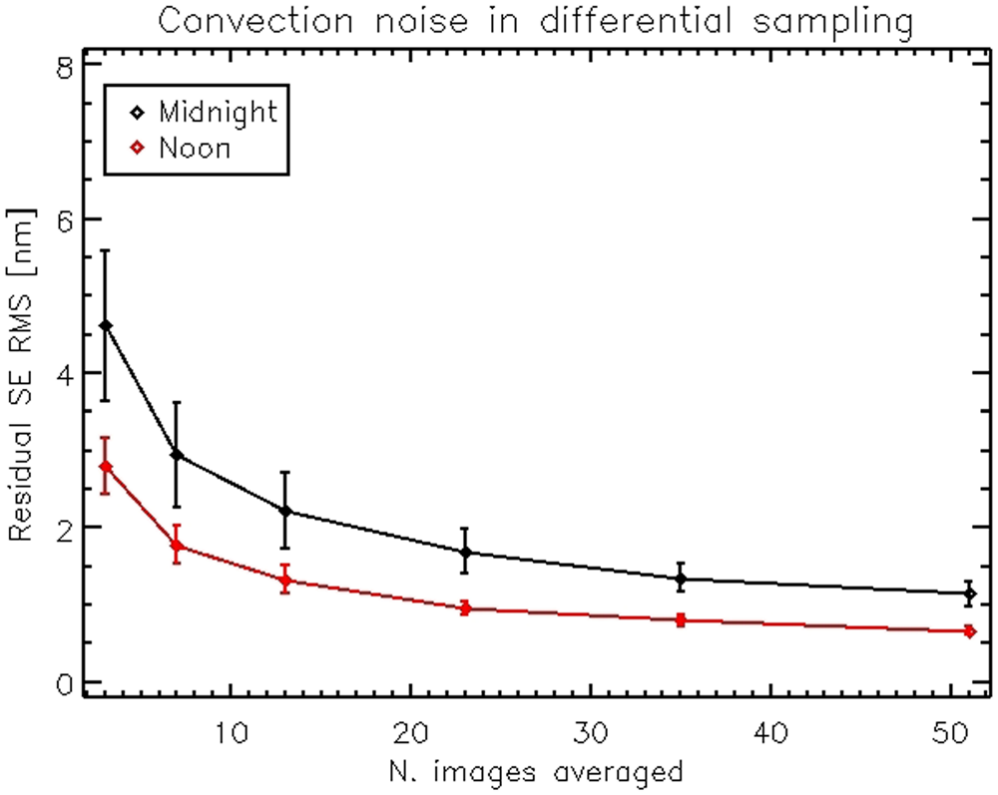
IF noise (surface error RMS) versus number of differential frames averaged.

**Figure 9:**
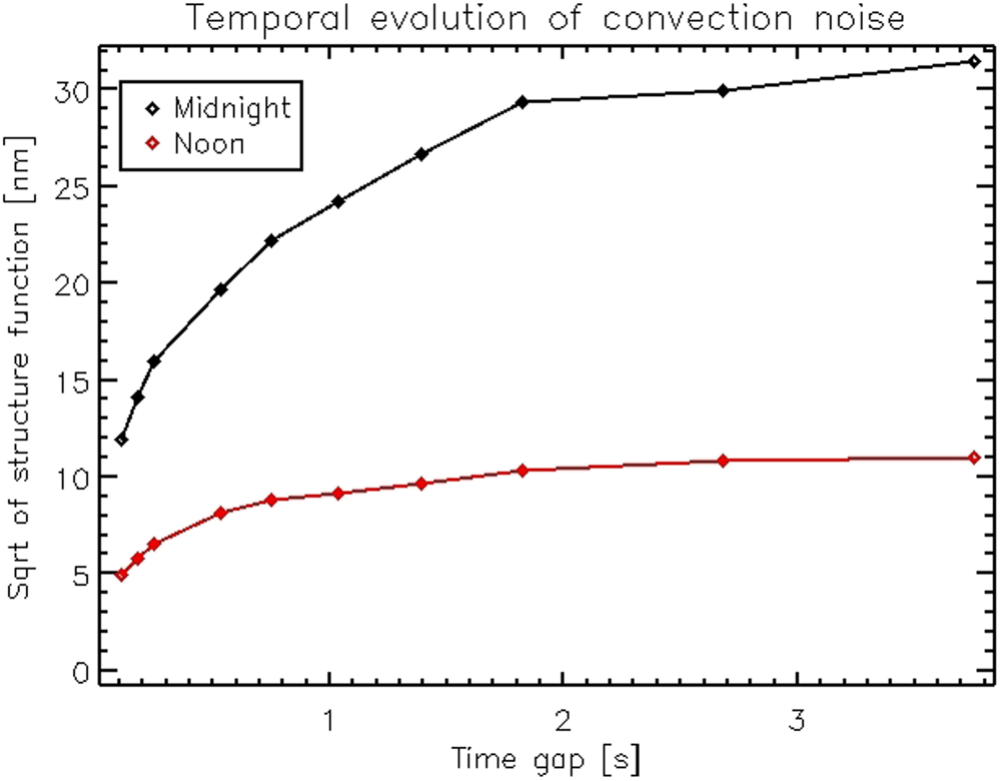
Temporal evolution of measurement noise due to air convection.

In Fig. 8 we show the expected noise for differential samplings versus number of differential frames averaged together. In Fig. 9 we show the result of the procedure for the estimation of the measurement accuracy (absolute measurements). The plot indicates the time coherence of convection noise and the expected variation of SE after a given time gap. Two realizations may be considered as un-correlated after 3 s (approximately twice the knee of the plot). Qualitatively, the errors associated with the average of two un-correlated frames may be computed as the asymptotic value of the plot divided by $\sqrt{2}$. As for the differential sampling noise, we observe that during night-time convection evolves faster towards a larger asymptotic value. More details on the computation are given in Sec. 4.3.

### Measurement of the mirror modes and fitting of the KL matrix

The final step of the optical calibration of an ASM is the fitting of the Karhunen-Loeve (KL) modes to run the AO loop with the WFS. The KL is an orthogonal matrix with the peculiar property to fit an atmospheric phase screen with the minimum number of modes, with respect to, for instance, Zernike or Fourier modes. For this reason, the KL expansion is widely adopted as a control matrix for AO. To this purpose, we sampled again the IF, averaging in this case 51 *push-pull* frames to significatively enhance the measurement SNR and improve the fitting. The modal amplitude was carefully tuned mode by mode. The trade-off criteria are summarized as follows:

- To maximize the amplitude for low order modes (0 to 50), those mostly contributing to KL tip/tilt and focus, in order to improve the fitting accuracy versus vibration noise;
- To lower the amplitude in order to avoid the fringes density saturation with loss of the interferometric signal. This applies especially for modes in the range 50 to 200 as they excite the areas at the TS edge.

For modes in the range 200 to 500, the amplitude is automatically thresholded by the maximum applicable actuator force. Considering the plot in Fig. 8, we estimated that the SNR achieved is larger than 50 for modes #0 to #200 and larger than 15 for modes #200 to #450. The SNR for mode #499 is 10. The tip-tilt noise from vibration (not considered within that plot) has been evaluated to be ≈2 nm RMS and assumed negligible for the low order modes, whose applied amplitude is 100 to 500 times larger. Tip/tilt was however removed from higher order modes before any further analysis.

**Figure 10:**
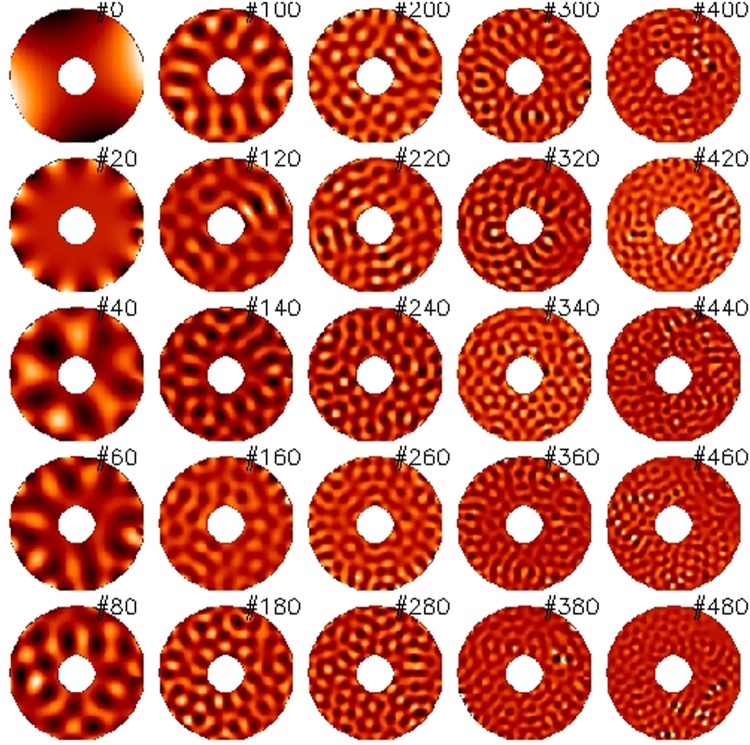
Surface map of the ASM mirror modes (sample).

**Figure 11:**
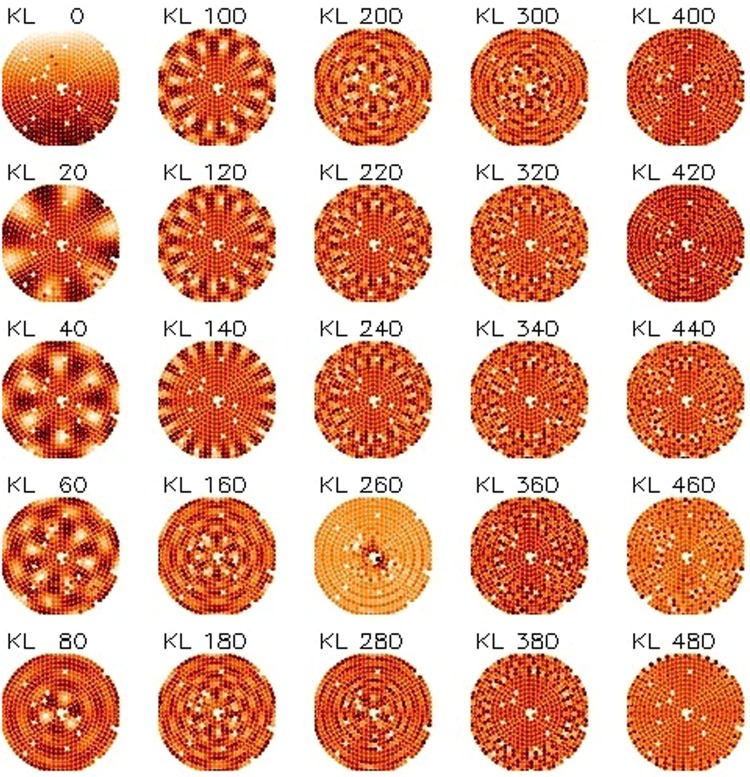
Actuator commands to produce the KL modes, as fitted from ASM mirror modes (sample).

The KL command matrix was computed by fitting the theoretical KL polynomials with the IF. The fitting process (described with more details in Sec. 4.4) included the slaving of those actuators lying below the central obstruction. Such process allows the contemporary optimization of the actuator force and of the fitting error when commanding actuators which cannot be directly controlled by the optical loop (in the present case, because of lack of visibility). In Figs 10 and 11 we present, respectively, a sample of the mirror IF and of the fitted KL commands.

### Verification of the slave-KL matrix

After fitting, we measured the slaved-KL command matrix with the same procedure as for the collection of the IF; we then tested the matrix by closing the optical loop with the interferometer. We performed a special test consisting in the flattening of a large (~10 *μ*m RMS SE) focus injected with a translation of the interferometer along the Z axis and registered the actuator forces for flattening. The requested negative focus command excited in fact the axially-symmetric KL modes, those mostly affected by the slaving procedure. The test was iterated several times and converged to the flattening result without saturation of the actuator force budget. The test demonstrated the effectiveness of the KL matrix.

### On-sky Demonstration

**Figure 12:**
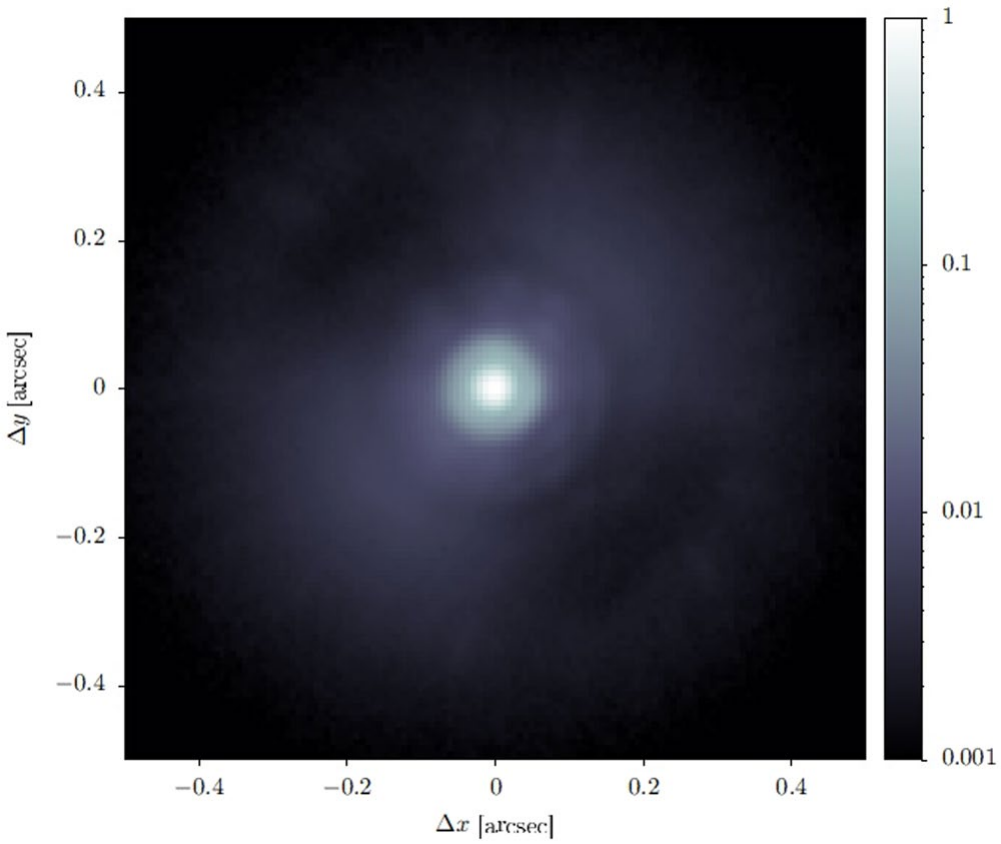
PSF (z’ band) obtained during the on-sky demonstration.

A brief on-sky demonstration was conducted to show that the new calibration works as intended. At the time of this demonstration, there were variable clouds, and seeing was 0.6” to 0.7” (LCO median seeing is 0.63”^10^). A bright I = 1 mag star was observed with the AO loop locked at 1000 Hz. Data were taken with the VisAO camera in the z’ filter (central wavelength 0.9 *μ*m), at 3.53 frames per second (the highest frame rate availabe in the full 1024 × 1024 pixel format). Images were dark subtracted, and registered and centered on the star, and then combined with a 5 *σ*-clipped mean. The resulting PSF measurement is shown in Fig. 12. The Strehl ratio was measured, relative to a theoretical Airy pattern for a 6.5 m telescope with a 29% central obstruction. The Strehl ratio of the PSF at 0.9 *μ*m in Fig. 12 is 28%. No significant photometric variability was noted in this data set, however the cloudy conditions make this Strehl ratio a lower limit. Due to the poor and variable conditions, direct comparison to pre-re-calibration results is difficult, but this is at least as good as expected for such conditions.

## Conclusion

We described the optical calibration procedure as performed on the ASM equipping the Magellan AO system at Las Campanas Observatory, Chile. The procedure is common for any large format, voice coil controlled adaptive mirrors, such as the LBT and VLT ones and the forthcoming adaptive mirrors for the E-ELT and GMT.

The concave ASM was measured in double pass by mean of a Twyman-Green interferometer and a retro-reflector, installed respectively at the mirror long focus and short focus. After the alignment and the preliminary shaping to reduce the local fringes density, we measured and corrected in close loop with the interferometer 500 mirror modes out of 555, achieving a final surface error of 22 nm RMS. Despite 30 not-working actuators, the optical quality is comparable to that measured during the system calibration and commissioning in 2011.

We used high SNR measurements of all the mirror modes to fit the KL command matrix requested for the AO loop; the fitting included a specific algorithm to manage the actuators obscured by the central obstruction. We tested the KL command matrix by closing the loop with the interferometer and we demonstrated the convergence and stability of the flattening procedure.

After the interferometer calibration, the ASM was ready for the MagAO calibration with the pyramid WFS and for closing the AO loop on sky.

## Methods and Setup

### Optical calibration procedure

The procedure for the optical calibration of large size deformable mirrors was developed at INAF-Osservatorio Astrofisico di Arcetri and adopted for the LBT^11^ and VLT^12^ ASM and for the E-ELT M4 prototype^13^.

The core is the interferometer sampling of the TS when an actuator command is applied; we compute the capacitive sensors gain by comparing the reading of the internal metrology with the actual displacement measured from the surface map produced by the interferometer. The optical setup to perform these measurements for the specific case of the MagAO ASM is described in Sec. 4.2. When the sensor gain is known, we can accurately produce a given optical shape by closing the internal loop on the corresponding command.

An ASM with *n* actuators may be controlled via its eigenmodes matrix *V*^14^, which is an *n* × *n* matrix ordered by mechanical stiffness (and inversely ordered by spatial scale); any actuator command may be written as a linear combination of these mirror modes, or vectors in *V*. The interferometer measurement of a mirror mode is also known as mode *influence function* (IF): the set of all the IFs is piled up in the *optical interaction matrix M*. Any mirror shape *s* measured by the interferometer may be decomposed as a linear combination of the modes in *M* plus a higher spatial scale residue.

The collection of *M* allows the calibration of the internal ASM metrology with an external absolute reference (the interferometer HeNe laser); when the system is calibrated, we control the actuators to produce a minimum surface error (SE) shape, or *flattening command*. Dealing with a large format ASM, two main issues arise: at first, the initial optical figure may be dramatically poor so that only a fraction is within the interferometer capture range (incomplete definition of *M*); then, large size optics require large testing facilities (or the telescope dome, as in the present case) which may affect the measurement of *M* or the flattening command with large noise due to vibrations and air convection. Such aspects are addressed in our procedure (see Sec. 4.3). The calibration operations may be summarized in the following steps:

1. preliminary shaping, i.e. the reduction of the local slope to allow a correct surface mapping over the whole pupil;
2. flattening process, consisting of:

- partial measurement of the eigenmodes 0 to i in V to assemble a partial Mi
- application of a partial flattening command ${c}_{i}=-\,{M}_{i}^{+}{w}_{i}$, where wi is the current surface map produced by the interferometer;
- verification measurement to check the flattening convergence at the step i;
- iteration of the previous steps from low to high spatial scales, by increasing the number of modes in step of (say) 50 modes, until the M is filled with all the degrees of freedom of the system.

3. characterization of the environment noise, to trade-off the sampling parameters for the final measurement of the IF;
4. final, high SNR measurement of all the IF;
5. slaving of innermost actuators and modal control basis (KL modes) fitting.

Because of tight schedule constraints, the process was aimed in this case at a) a fast calibration of the flattening command with a SE within the pyramid WFS capture range and b) the high SNR measurement of the IF for the modal control basis fitting. No extra time was allocated for further optimization of the flattening command and its verification (e.g. with a long time-averaging to reduce the convection noise).

### Optical setup at the Clay telescope

**Figure 13:**
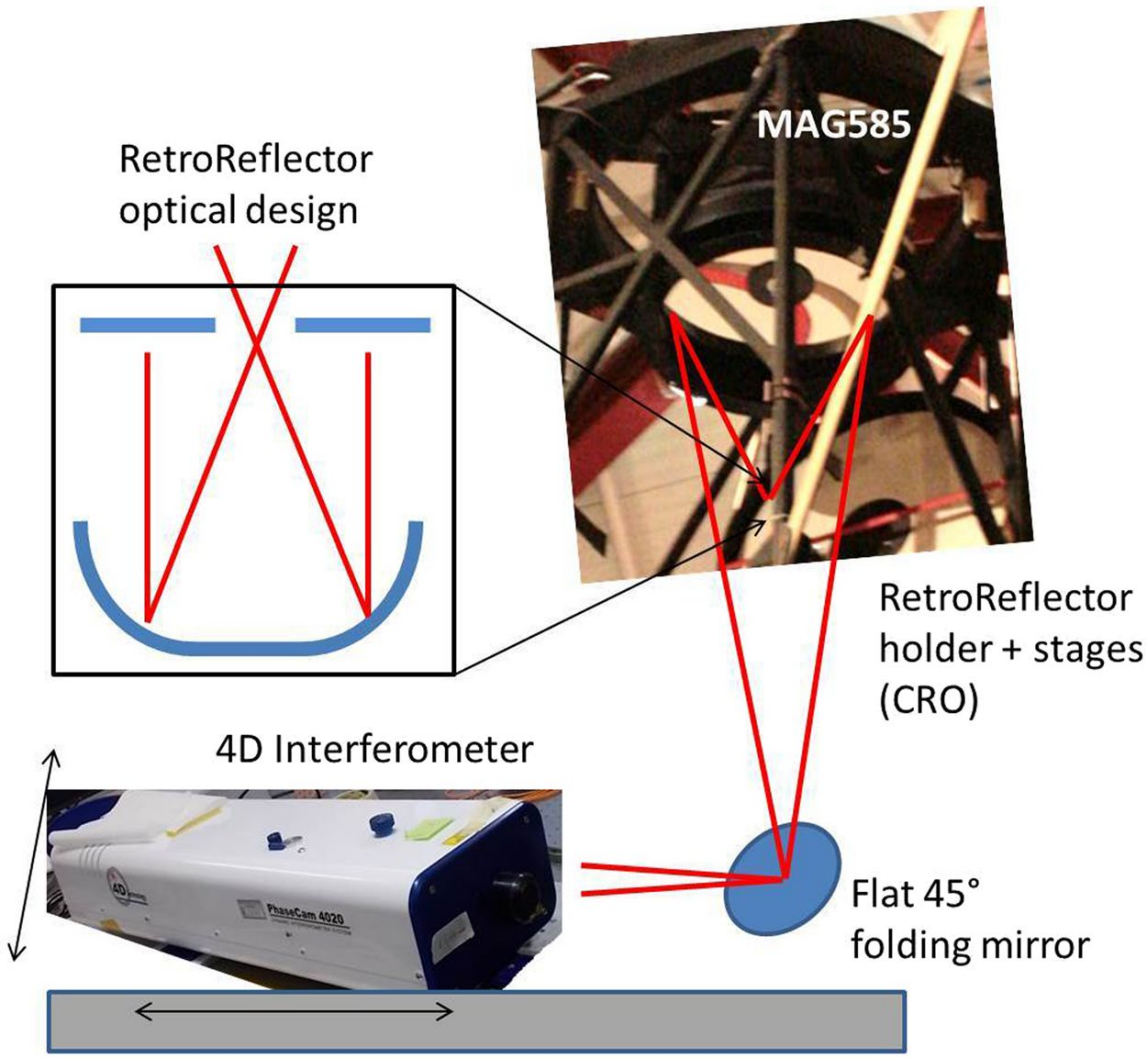
Scheme of the optical setup for the interferometer calibration of the ASM.

The MagAO ASM is an concave, elliptical mirror which conjugates a short focus at 1 m focal distance with a long one at 15 m. The mirror shape is measured in double pass with the interferometer placed at the long focus and a retro-reflector (RR) placed at the short focus. The RR is composed of a flat annular mirror at the focus of a fast F/1 parabola, matching both focus and pupil of reflected beam to those of the illumination one. An optical scheme of the RR within a sketch of the test layout is given in Fig. 13.

The optical measurements have been carried out with a 4D-Technology, PhaseCam 4020 interferometer. It is a Twyman-Green system offering dynamic interferometry, i.e. a single frame is requested to produce a phase map. Such feature is very useful to allow testing in laboratories with large vibrations and air convection. As the capturing time is as short as tens of micro-seconds, the noise pattern is frozen on the image with no impact on the phase-unwrapping process. The interferometer bench was mounted on the telescope rotator via a platform interface, fitted with a X-Z stage for the coarse alignment and a flat folding mirror to steer the beam toward the secondary. The fold mirror is at the system focus thus allowing a fine pupil centering on the CCD frame. The system was preliminarily aligned by means of X-Y shifts of the RR assembly and tip-tilt of the M2 and RR together; the telescope M3 was in the end rotated to adjust the residual tilt. The initial alignment achieved suffered for a large (~5 *μ*m PtV surface) coma offset, which was compensated with a coma shape applied by the actuators. The command offset was then relaxed at the end of the procedure.

### Characterization of dome noise

The dome was kept closed (i.e. no ventilation) for the entire calibration run. The environmental noise was measured both in terms of vibrations and air convection at different times during the day (e.g. noon, night, sunset) and some results are presented in Sec. 2.3. The data sampling consisted in the collection of a set of 4000 images at the interferometer frame rate (25 Hz), with no command applied to the mirror. The dataset was then processed according to two pipelines to evaluate the noise associated to differential and absolute measurements.

For differential sampling we processed the frames sequence to evaluate the resulting surface map after averaging *n* frame, as

3 $${w}_{n}=\frac{1}{n}\sum _{i=1}^{n}({(-1)}^{i+1}{\varphi }_{i+1}+{(-1)}^{i}{\varphi }_{i})$$

Such algorithm is the same as for the measurement of the IF: in the present case, no differential command was applied to the mirror so that the difference *ϕ*_*i* + 1_ − *ϕi* is a realization of the instantaneous differential noise at the sampling frequency. For the measurement of convection noise, tip, tilt and focus were removed from each differential frame *w*_*n*_; separately, tip and tilt were measured for the evaluation of vibration noise. The RMS of the *w*_*n*_ is taken as the expected surface error when averaging *n* frames. From these results, we might identify the suitable combination of command amplitude and value of *n* to achieve a given SNR.

For the case of absolute measurements, we evaluated the expected contribution of convection noise by computing the structure function of the dataset. Following Hardy^15^, we define the structure function *D*_*ϕ*_(*τ*) as

4 $${D}_{\varphi }(\tau )= < \,{({\varphi }_{p}({t}_{i}+\tau )-{\varphi }_{p}({t}_{i}))}^{2}\, > =\frac{1}{npix}\frac{1}{\eta }\sum _{p=1}^{npix}\sum _{i=1}^{\eta }{({\varphi }_{p}({t}_{i}+\tau )-{\varphi }_{p}({t}_{i}))}^{2}$$

where *ϕ*_*p*_(*t*_*i*_) is one of the *η* interferometer measurements (being *p* the pixel index) and *τ* is a time gap. Here, we added without any loss of generality the *ensemble* average by computing the mean value of the pixel, after verifying that the realizations of the *D*_*ϕ*_(*τ*) are pixel-wise homogeneous.

Under the assumption that *ϕ*(*t*) is stationary and with zero mean value, we may introduce the correlation function *B*(*τ*) to get

5 $${B}_{\varphi }(\tau )= < \,\varphi (t+\tau )\varphi (t) > $$

6 $${D}_{\varphi }(\tau )=\mathrm{2(}{B}_{\varphi }\mathrm{(0)}-{B}_{\varphi }(\tau ))$$

7 $${D}_{\varphi }(\infty )=2{B}_{\varphi }\mathrm{(0)}=2{\sigma }^{2}(f)$$

where *σ*^2^ is the mean square value of *ϕ*(*t*) and *D*_*ϕ*_(∞) is the asymptotic value of the structure function. *B*_*ϕ*_(∞) is assumed to be 0. By computing the square root of *D* we obtain in the end:

8 $${S}_{\varphi }(\tau )=\sqrt{{D}_{\varphi }(\tau )}$$

9 $${S}_{\varphi }(\infty )=\sqrt{2{B}_{\varphi }\mathrm{(0)}}=\sqrt{2}\sigma (f)$$

The plots in Fig. 9 are the measurement of *S*_*ϕ*_(*τ*) whose units are [m]: its asymptotic value is $\sqrt{2}$ the expected noise (RMS) associated with the sampling of a single interferometric measurements. The number of frames *n*_*p*_ requested to achieve a measurement noise *ε* may be then computed as

10 $${n}_{p}={(\sigma /\varepsilon )}^{2}=\frac{1}{2}{({S}_{\varphi }(\infty )/\varepsilon )}^{2}$$

under the assumption that the frames are un-correlated. Two frames may be considered un-correlated if they are sampled after a time gap larger than their coherence time, estimated from the dataset as twice the abscissa of the knee of the curve. A robust time averaging requests therefore a slow sampling of (say) tens of frames over a time span of several minutes. Such sampling was not possible due to schedule reasons, so that our flattening realization is affected by a (typical) convection noise of 15 to 20 nm RMS, as discussed in Sec. 2.2. Such residual offset has been accepted as it is well within the requirements of optical quality for the PWFS (see also Sec. 4).

### Slaving and modal control basis fitting

The three innermost actuator rings (a total of 45 actuators) lie behind the shadow of the Clay telescope’s central obscuration. The control of a large number of actuators outside the optical pupil represented a challenge that required a new control strategy because these un-illuminated actuators could not be simply disabled. Disabling an actuator prevents it from applying any force on the shell leaving it free to “float” at that particular location. This is in fact an ideal solution for individual mis-functioning actuators for which neighboring ones can take over the weight per actuator of the shell. The control bandwidth of those neighboring actuators (which is related to the damping per unit mass) will be just slightly reduced. On the contrary, large clusters of disabled actuators compromise the controllability of the neighboring ones leading to instabilities in the overall ASM control.

Un-illuminated actuators in rings #1 to #3 had to be controlled and so a *slaving* strategy was developed. The original strategy implemented in 2012 consisted in a transformation of *V* that ensured that any commands applied to *slaved* actuators would require null force on them. This strategy provided an acceptable on-sky performance under good seeing conditions, but caused force saturation on the neighboring actuators (rings #4 and #5) when operating under stronger turbulence. The slaving strategy developed for the 2016 MagAO re-tuning campaign was designed to apply a command vector to the slaved actuators that minimized the force RMS of the whole set of “masters/slaves” actuators (i.e. rings #1 to #5). This alternative slaving criterion performed much better under all seeing conditions because slaves effectively help masters to control the shell in proximity of the central obscuration.

The shape of the *slaved* IF can be easily computed from the measured *optical interaction matrix M* applying the same transformation to it, without the need to measure the *slaved* IF again with the interferometer. The set of high SNR *slaved* IF derived in this way was then used to fit the AO control modes, which are based on the Karhunen-Loève (KL) modal basis. KL modes provide the highest atmospheric disturbance rejection for a given number of corrected modes, with respect to other possible modes such as Zernike or Fourier modes.

The KL fitting procedure is the same we developed for the LBT AO system. In brief, we computed a set of theoretical tilt-free KL functions combined with the Zernike tilt functions to form an orthogonal set on the Magellan pupil (central obscuration is taken into account). Then, these modes were projected onto the set of slaved IF following a conventional least-squares approach. Finally, because the orthonormality of the fitted KL modes is no longer guaranteed, these modes were re-orthonormalized through the Gram-Schmidt process. The final product of these computations is the so-called *modes-to-commands matrix* containing the ASM commands that generate the desired controlled modes.
